# Supplementary figures and images for: Small-molecule activation of TFEB alleviates Niemann–Pick disease type C via promoting lysosomal exocytosis and biogenesis
Source: eLife. 2025 Apr 4;13:RP103137. doi: 10.7554/eLife.103137 (PMC11970905; doi:10.7554/eLife.103137)

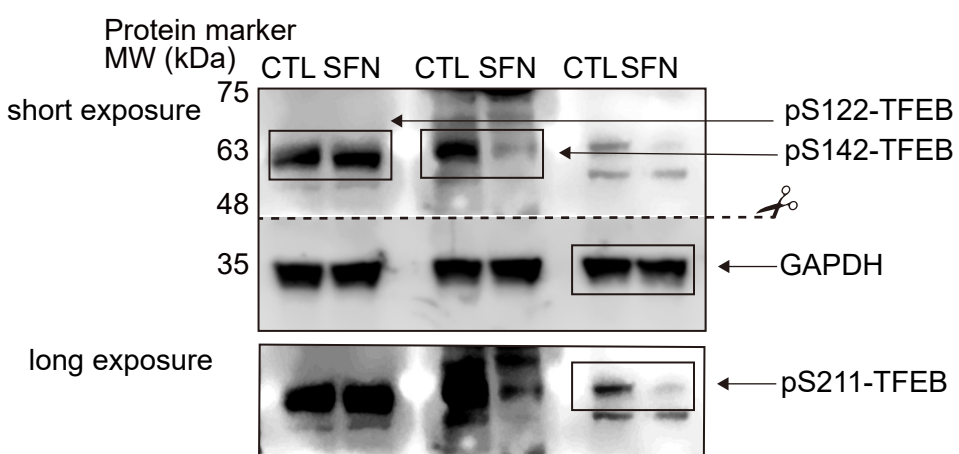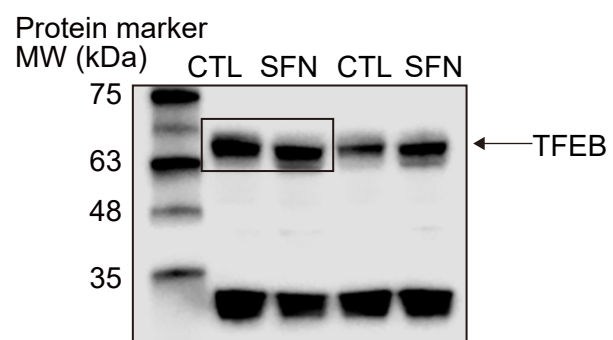

Supplement: Figure 1—source data 1. [file elife-103137-fig1-data1.zip › Figure 1–Source Data 1/Figure 1I–Source Data 1.pdf]

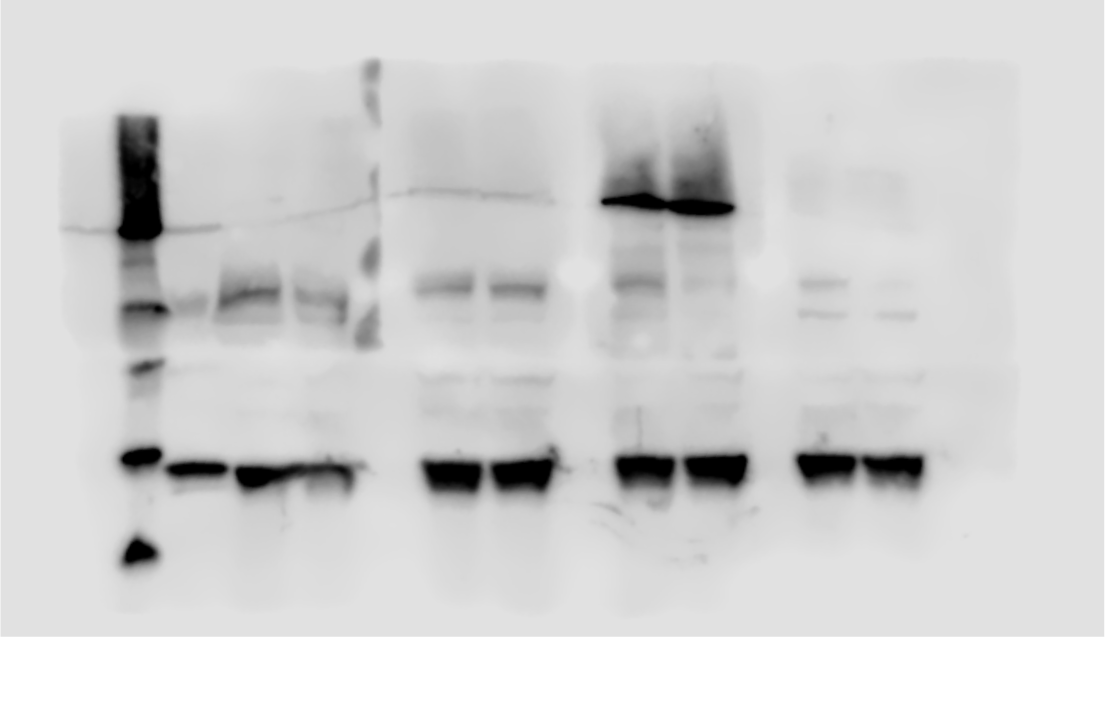

Supplement: Figure 1—source data 2. [file elife-103137-fig1-data2.zip › Figure 1–Source Data 2/Figure 1I–Source Data 2-1.tif]

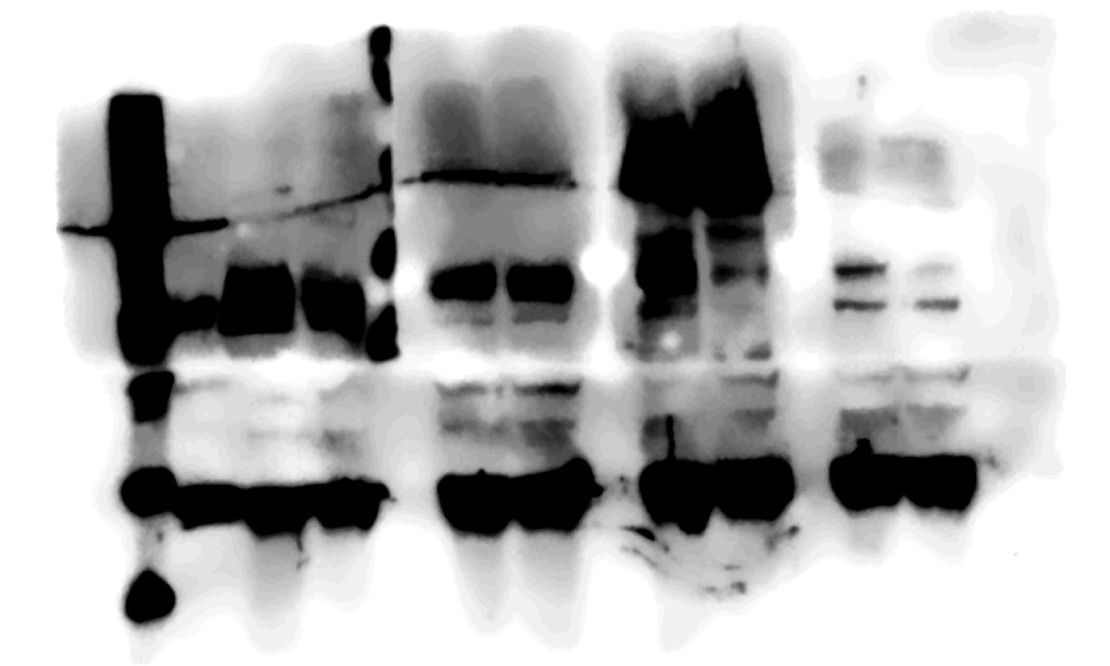

Supplement: Figure 1—source data 2. [file elife-103137-fig1-data2.zip › Figure 1–Source Data 2/Figure 1I–Source Data 2-2.tif]

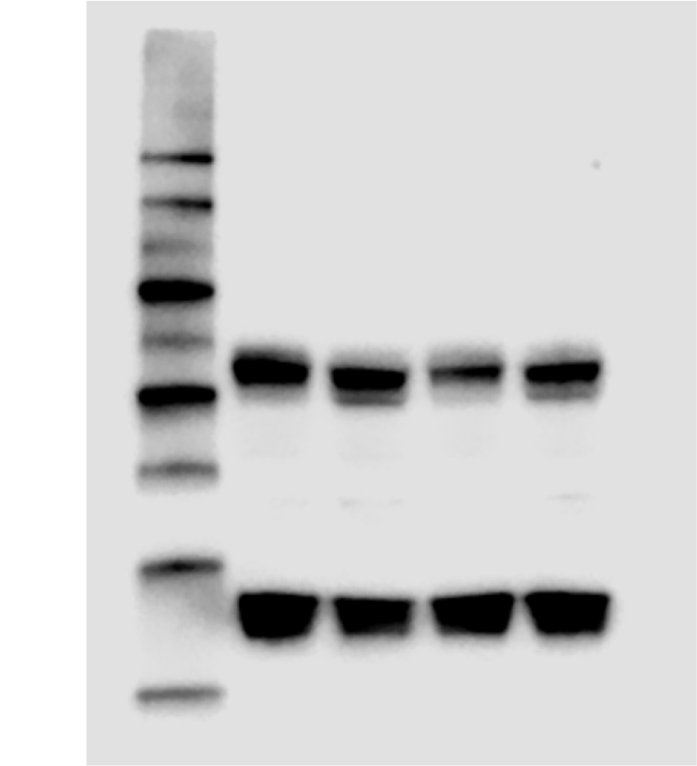

Supplement: Figure 1—source data 2. [file elife-103137-fig1-data2.zip › Figure 1–Source Data 2/Figure 1I–Source Data 2-3.tif]

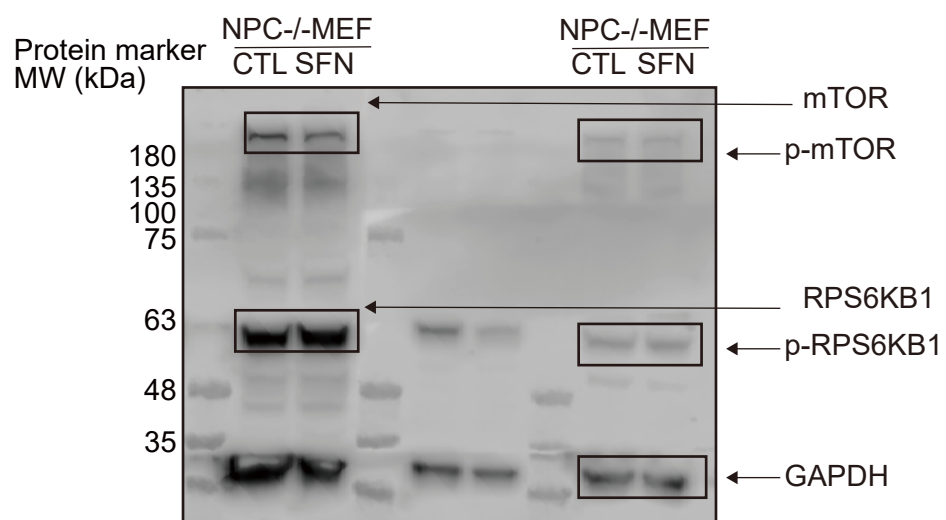

Supplement: Figure 1—figure supplement 4—source data 1. [file elife-103137-fig1-figsupp4-data1.zip › Figure 1–figure supplement 4–Source Data 1/Figure 1–figure Supplement 4–Source Data 1.pdf]

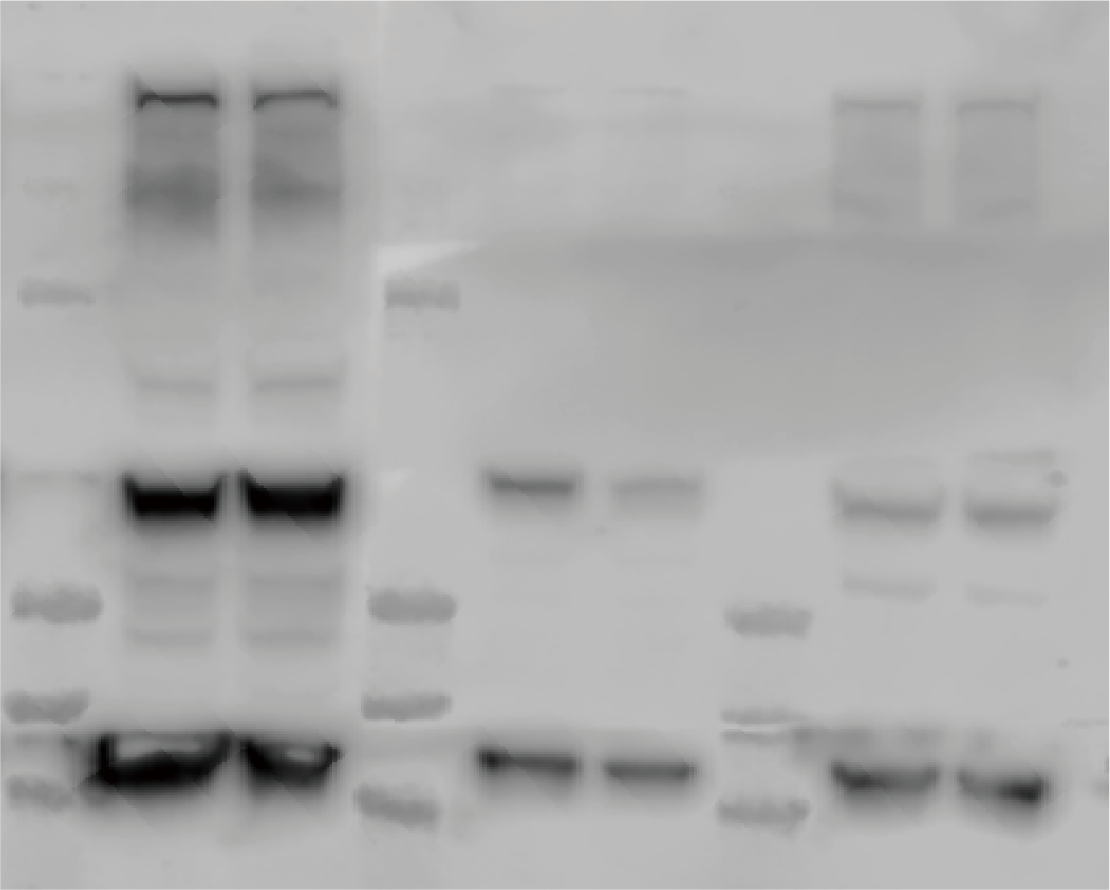

Supplement: Figure 1—figure supplement 4—source data 2. [file elife-103137-fig1-figsupp4-data2.zip › Figure 1–figure supplement 4–Source Data 2/Figure 1–figure supplement 4–Source Data 2.tif]

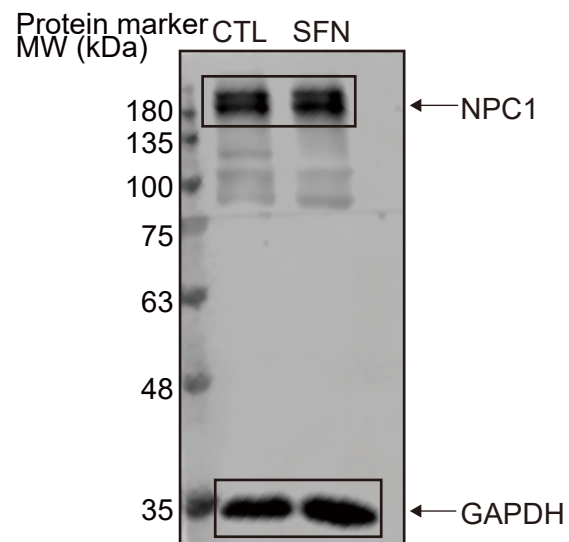

Supplement: Figure 1—figure supplement 5—source data 1. [file elife-103137-fig1-figsupp5-data1.zip › Figure 1–figure supplement 5–Source Data 1/Figure 1–figure Supplement 5–Source Data 1.pdf]

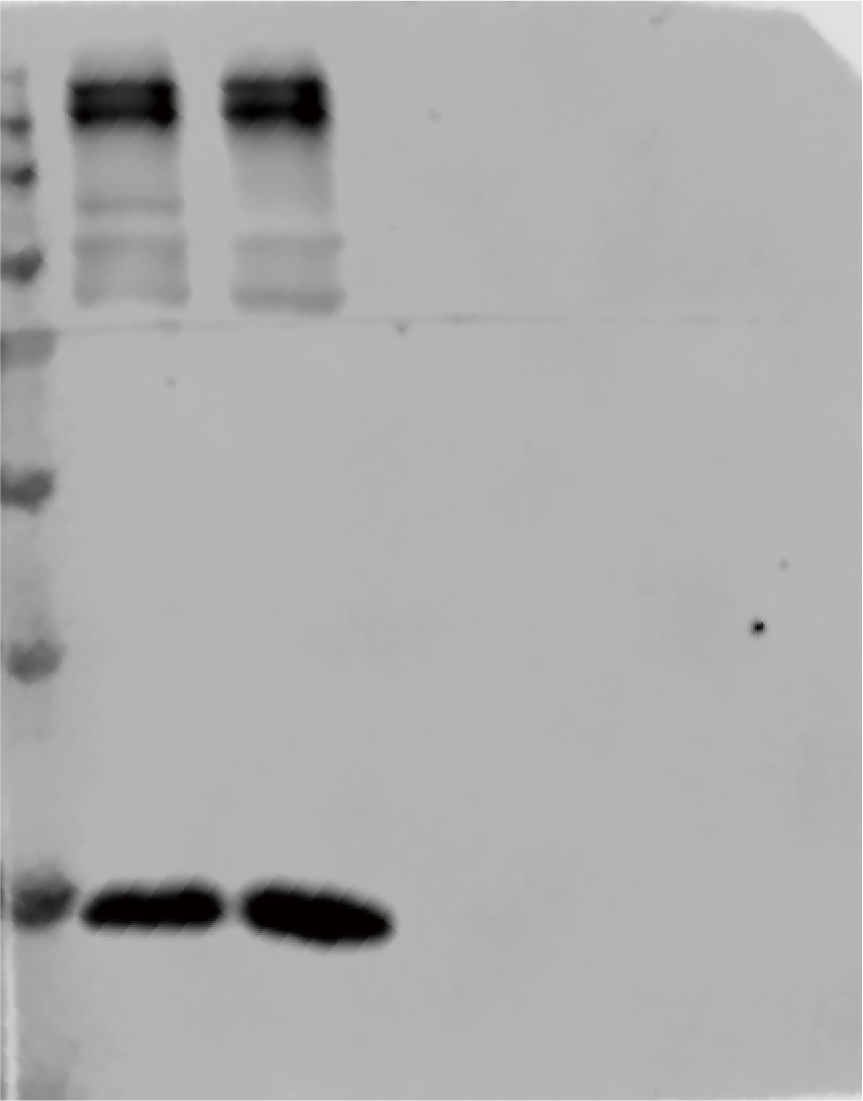

Supplement: Figure 1—figure supplement 5—source data 2. [file elife-103137-fig1-figsupp5-data2.zip › Figure 1–figure supplement 5–Source Data 2/Figure 1–figure supplement 5–Source Data 2.tif]

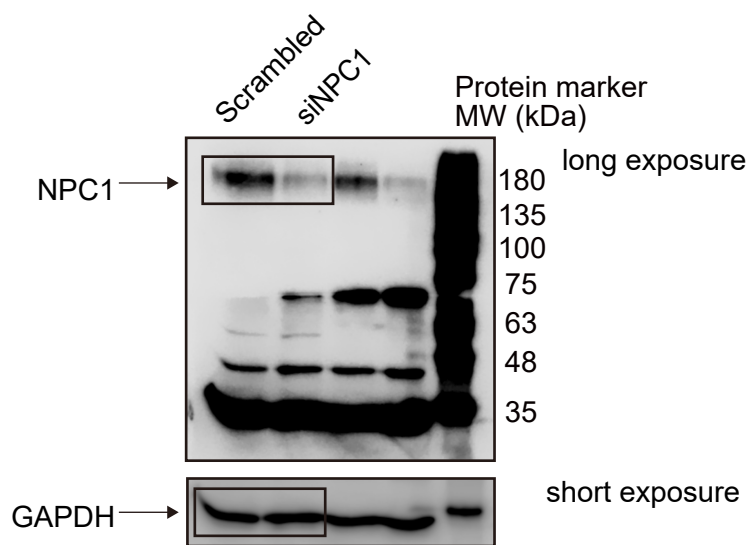

Supplement: Figure 2—source data 1. [file elife-103137-fig2-data1.zip › Figure 2–Source Data 1/Figure 2A–Source Data 1.pdf]

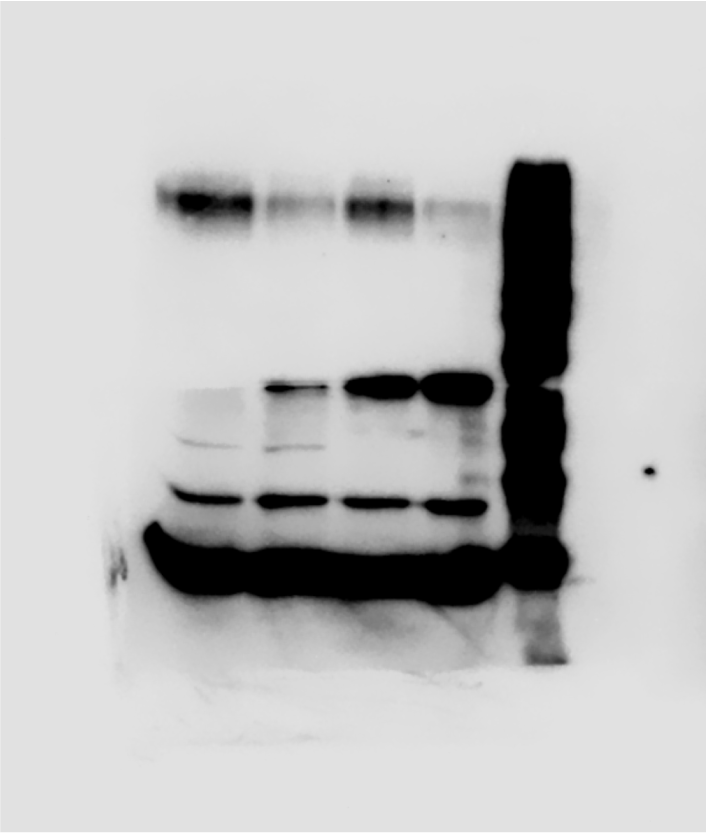

Supplement: Figure 2—source data 2. [file elife-103137-fig2-data2.zip › Figure 2–Source Data 2/Figure 2A–Source Data 2-1.tif]

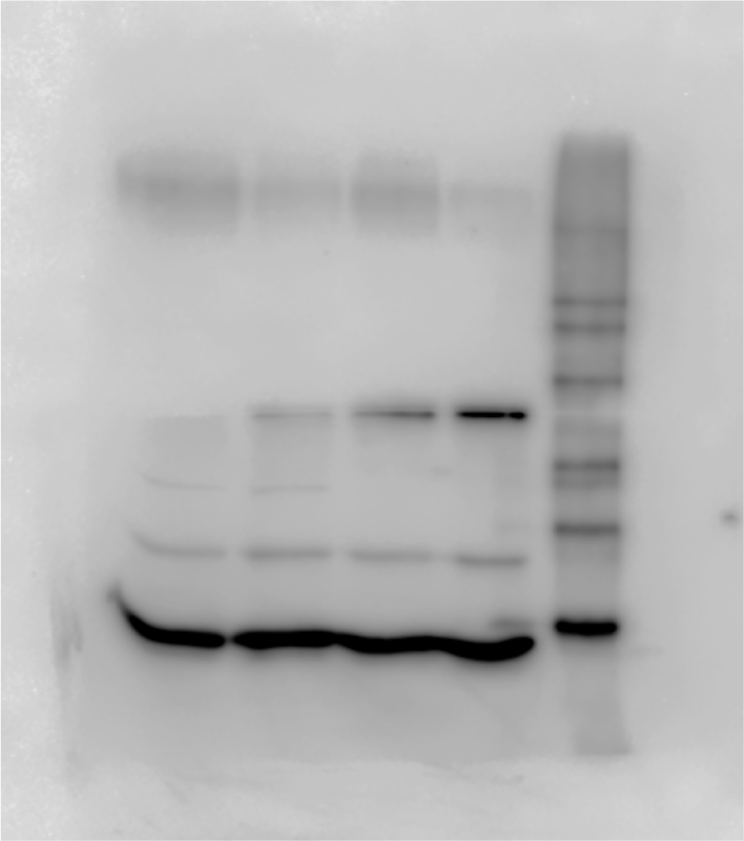

Supplement: Figure 2—source data 2. [file elife-103137-fig2-data2.zip › Figure 2–Source Data 2/Figure 2A–Source Data 2-2.tif]

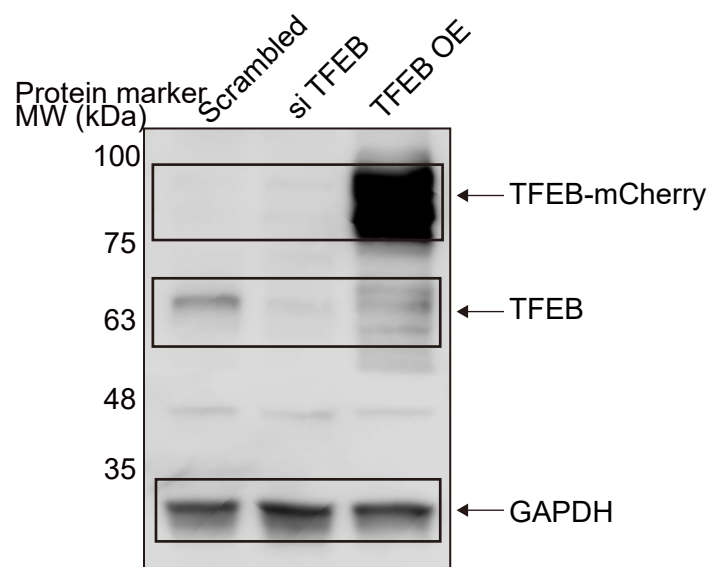

Supplement: Figure 3—source data 1. [file elife-103137-fig3-data1.zip › Figure 3–Source Data 1/Figure 3A–Source Data 1.pdf]

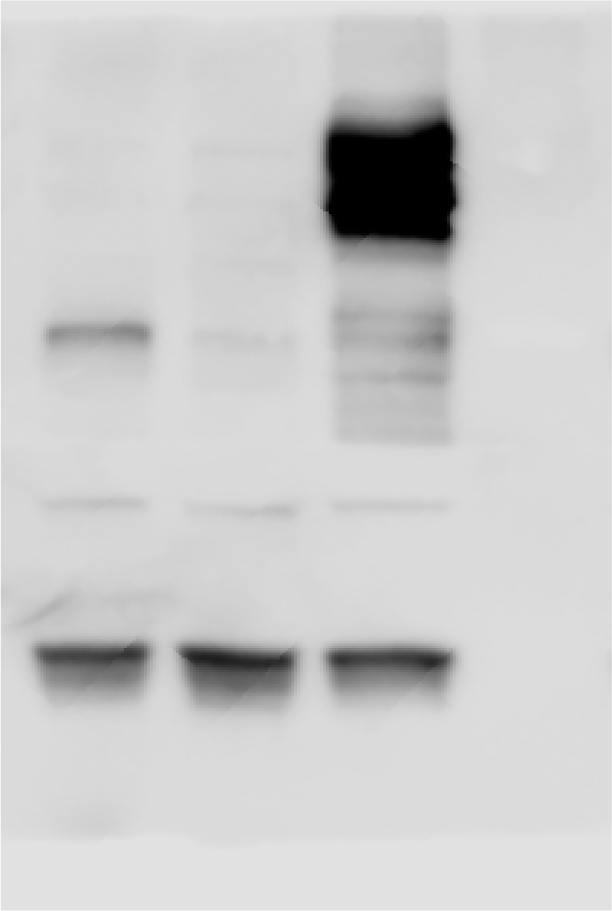

Supplement: Figure 3—source data 2. [file elife-103137-fig3-data2.zip › Figure 3–Source Data 2/Figure 3A–Source Data 2.tif]

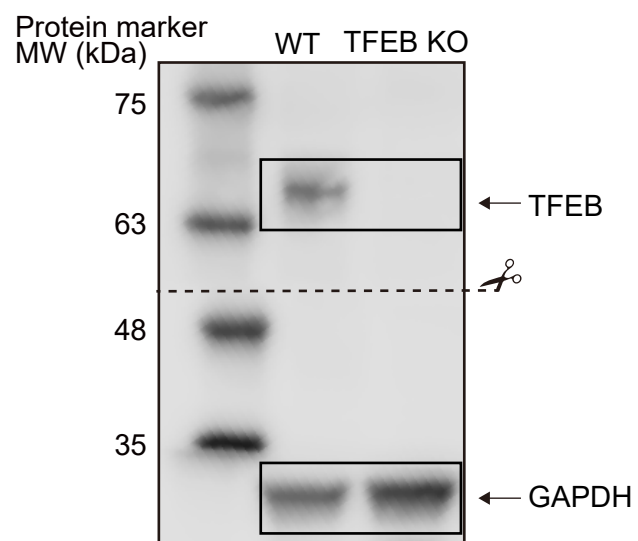

Supplement: Figure 3—source data 3. [file elife-103137-fig3-data3.zip › Figure 3–Source Data 3/Figure 3D–Source Data 3.pdf]

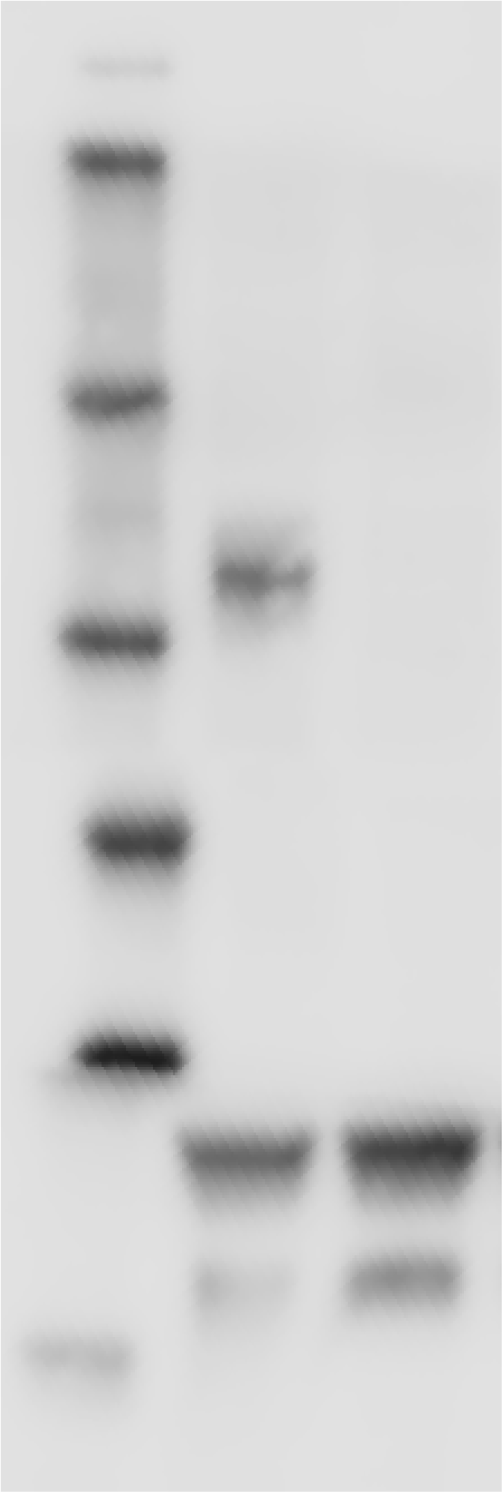

Supplement: Figure 3—source data 4. [file elife-103137-fig3-data4.zip › Figure 3–Source Data 4/Figure 3D–Source Data 4.tif]

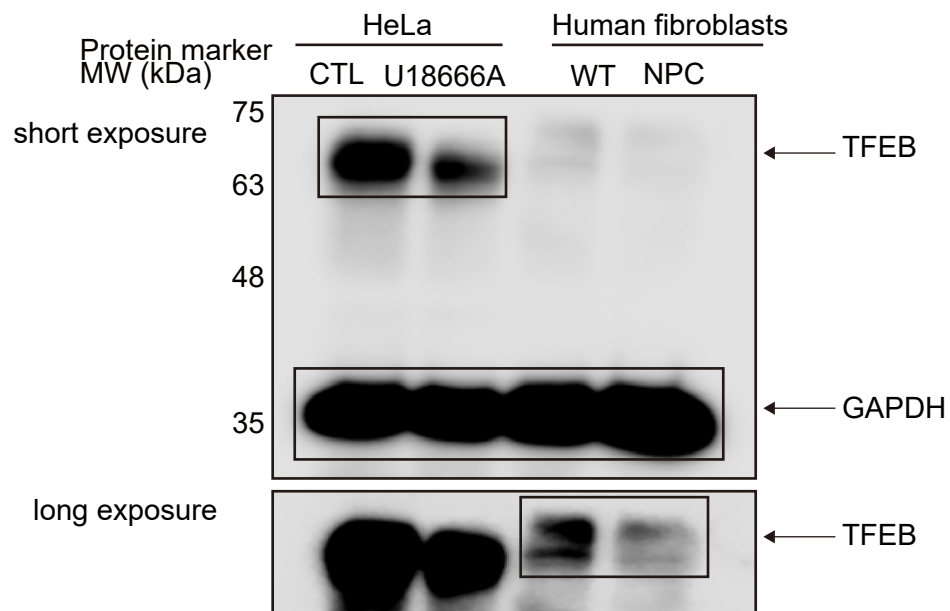

Supplement: Figure 4—figure supplement 3—source data 1. [file elife-103137-fig4-figsupp3-data1.zip › Figure 4–figure supplement 3–Source Data 1/Figure 4 –figure supplement 3–Source Data 1.pdf]

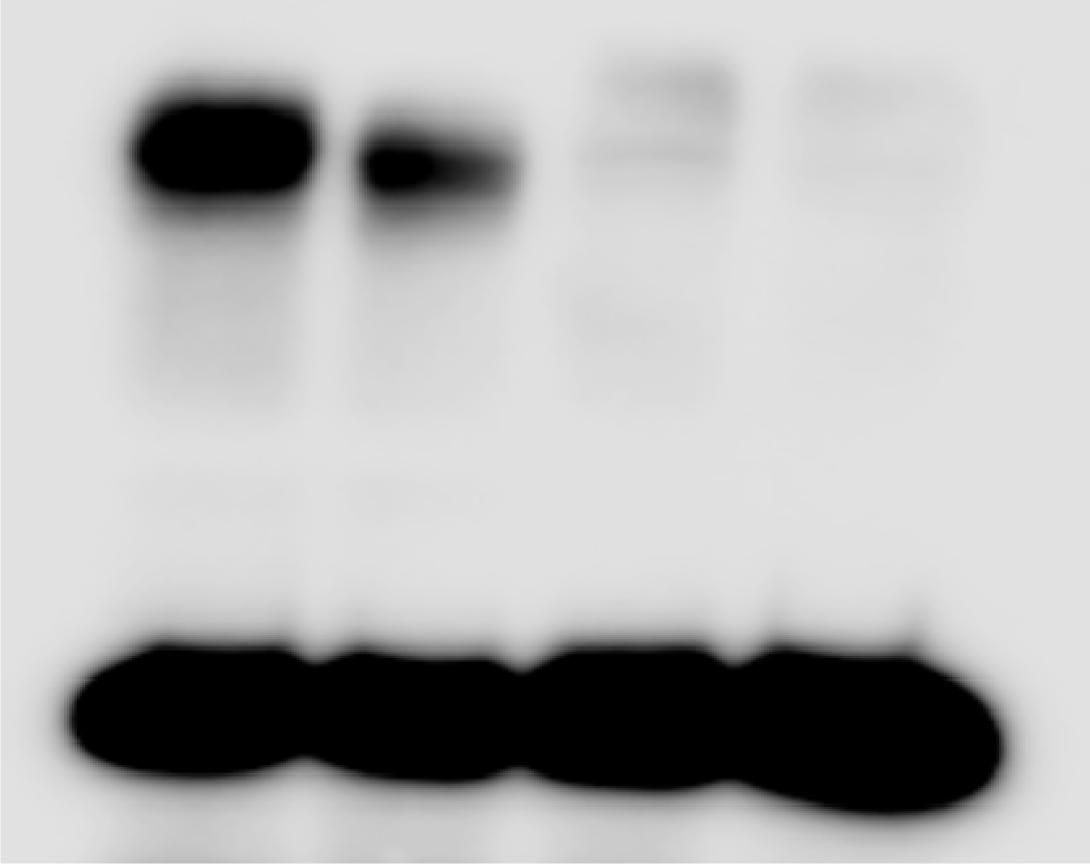

Supplement: Figure 4—figure supplement 3—source data 2. [file elife-103137-fig4-figsupp3-data2.zip › Figure 4–figure supplement 3–Source Data 2/Figure 4–figure supplement 3–Source Data 2-1.tif]

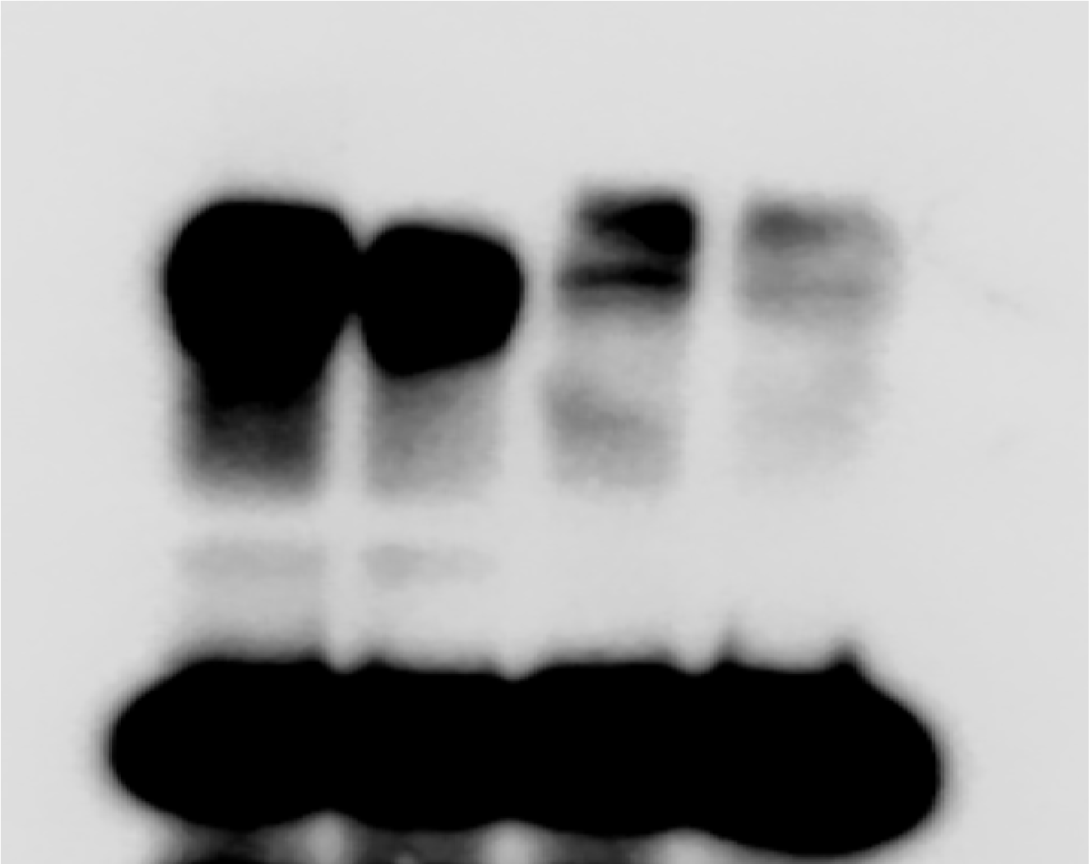

Supplement: Figure 4—figure supplement 3—source data 2. [file elife-103137-fig4-figsupp3-data2.zip › Figure 4–figure supplement 3–Source Data 2/Figure 4–figure supplement 3–Source Data 2-2.tif]

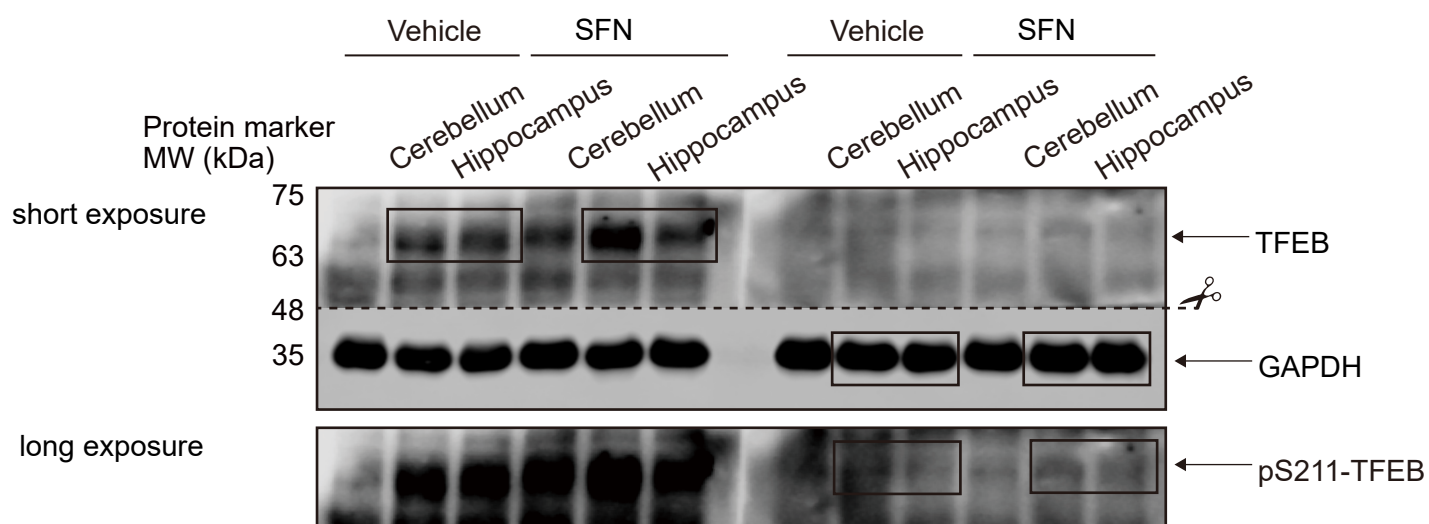

Supplement: Figure 6—source data 1. [file elife-103137-fig6-data1.zip › Figure 6–Source Data 1/Figure 6A–Source Data 1.pdf]

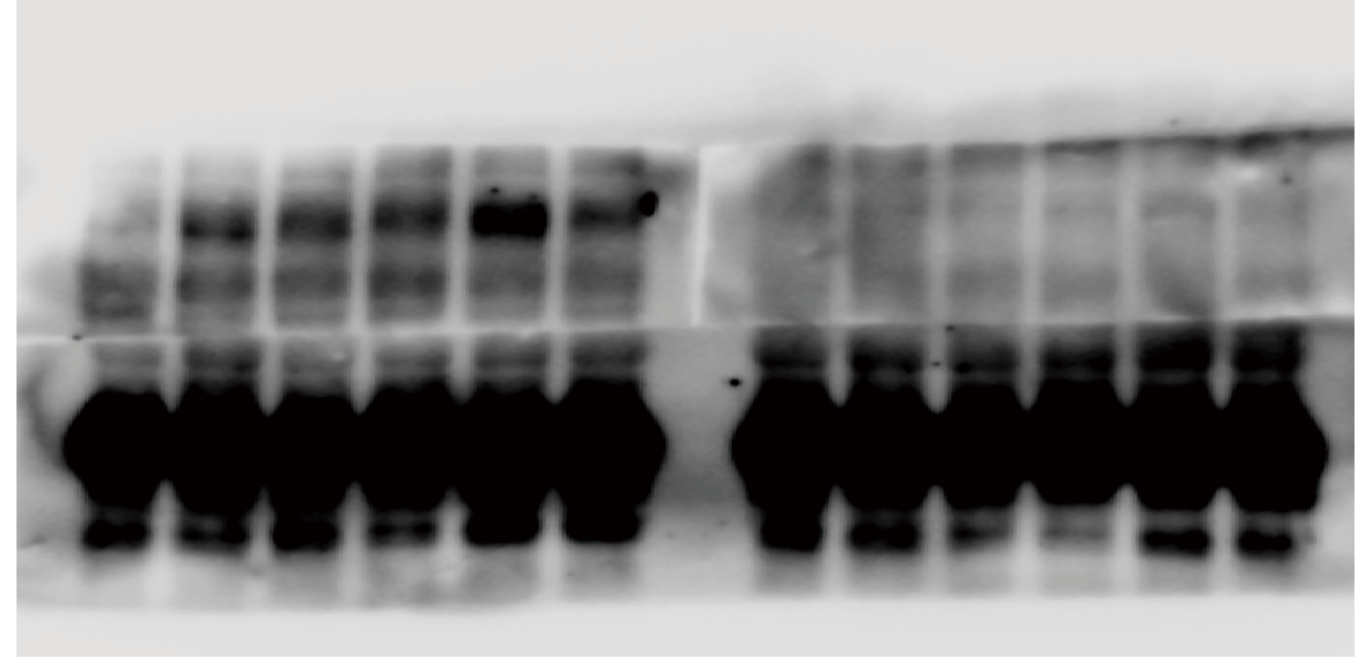

Supplement: Figure 6—source data 2. [file elife-103137-fig6-data2.zip › Figure 6–Source Data 2/Figure 6A–Source Data 2-1.tif]

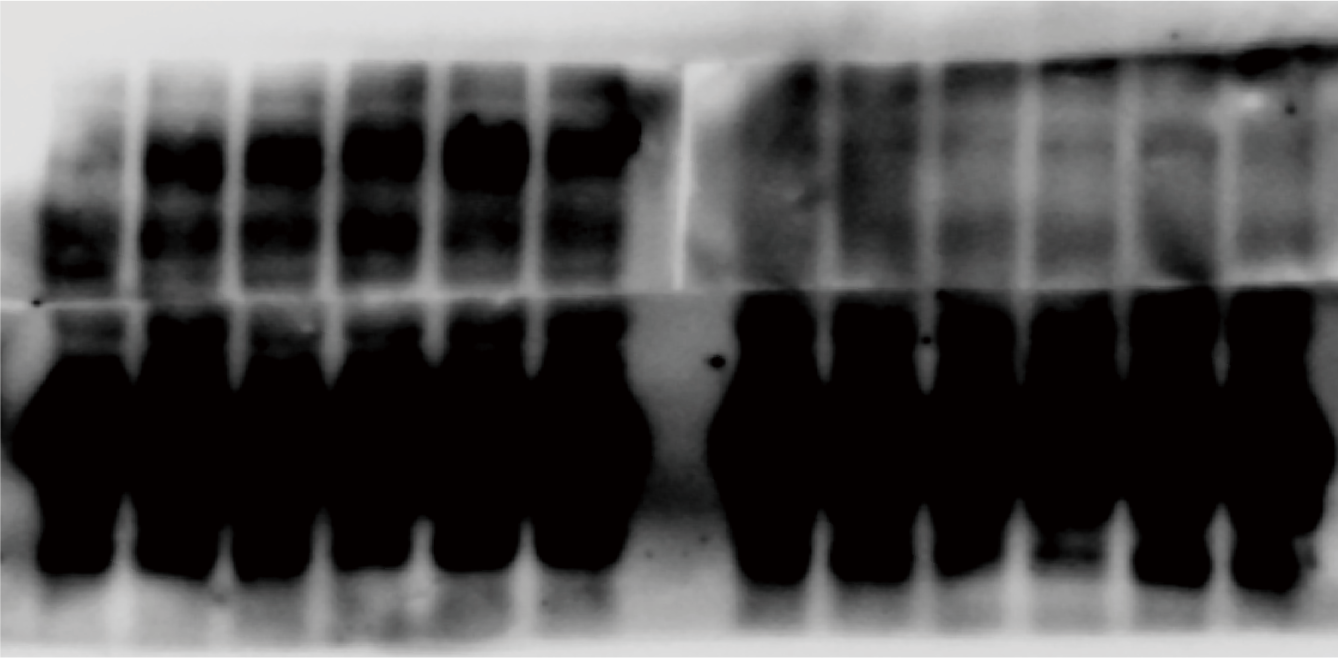

Supplement: Figure 6—source data 2. [file elife-103137-fig6-data2.zip › Figure 6–Source Data 2/Figure 6A–Source Data 2-2.tif]

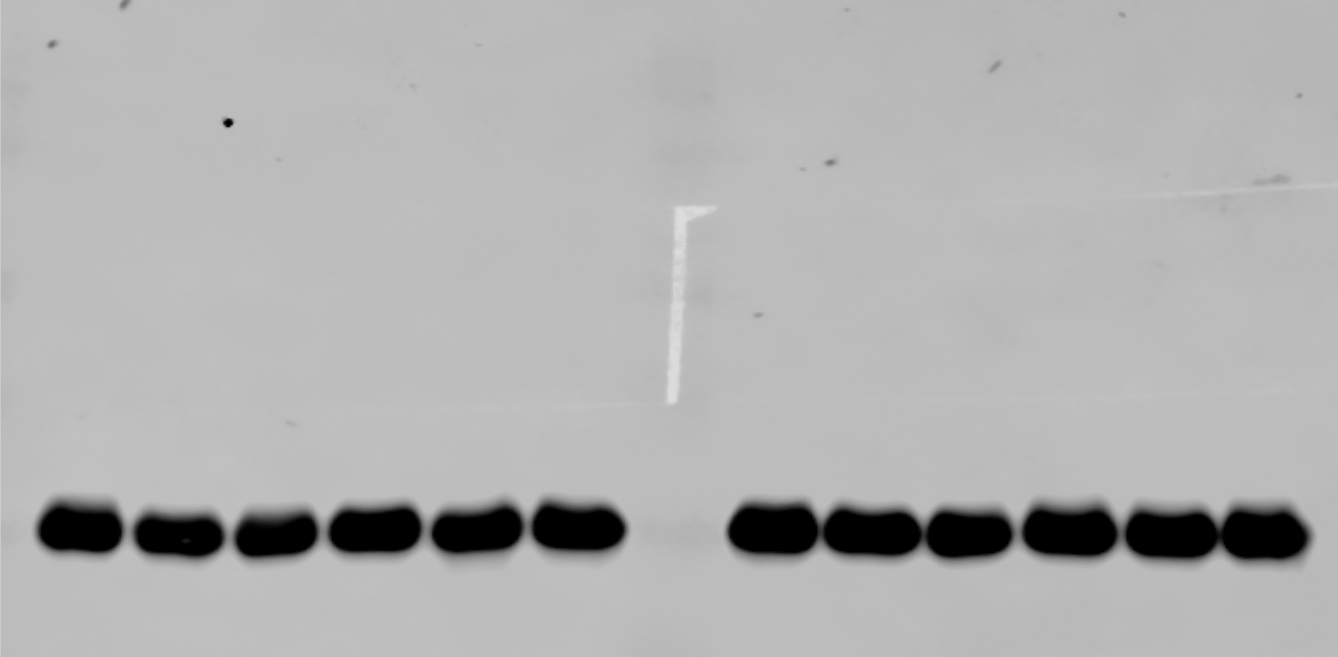

Supplement: Figure 6—source data 2. [file elife-103137-fig6-data2.zip › Figure 6–Source Data 2/Figure 6A–Source Data 2-3.tif]
